# The Influence of Slag/Fly Ash Ratio and Sodium Silicate Modulus on the Properties of 1-3-2 Alkali-Based Piezoelectric Composite

**DOI:** 10.3390/ma15031150

**Published:** 2022-02-02

**Authors:** Jiayi Cai, Zhanyi Peng, Ruohong Zhao, An Xu, Xinyu Zhou

**Affiliations:** 1 Research Center for Wind Engineering and Engineering Vibration, Guangzhou University, Guangzhou 510006, China; 2111816072@e.gzhu.edu.cn (J.C.); xuan@gzhu.edu.cn (A.X.); 2111716281@e.gzhu.edu.cn (X.Z.); 2 Department of Architecture, Kanagawa University, Yokohama 221-8686, Japan; 3 Department of Civil Engineering, Guangzhou University, Guangzhou 510006, China; 2111716202@e.gzhu.edu.cn

**Keywords:** 1-3-2 piezoelectric composite, AAFS, sodium silicate modulus, piezoelectric properties

## Abstract

In this paper, a comprehensive experimental investigation on the effect of the slag-to-fly ash ratio (hereafter referred to as SL/FA) and sodium silicate modulus on the properties of a 1-3-2 piezoelectric composite was carried out. The influence of the SL/FA ratio on various properties was initially investigated. Compared with other specimens, specimens with SL/FA = 40%:60% had the highest electromechanical coupling coefficient (Kt = 77.67%, Kp = 71%). Therefore, the specimen with SL/FA = 40%:60% was chosen to explore the effect of the sodium silicate modulus. Additionally, the specimen with SL/FA = 40%:60% and a sodium silicate modulus of 1.3 had the best electromechanical conversion efficiency with Kt = 75.68% and Kp = 75.95%. The 1-3-2 alkali-based piezoelectric composite proved to have the characteristics of a low cost, optimal piezoelectric and mechanical properties, higher tunability, and better compatibility with concrete. It is a potential alternative to cement-based piezoelectric composites and may be widely utilized to monitor the health of concrete structures.

## 1. Introduction

The building of bridges and highways requires the use of a considerable volume of concrete. Large-volume concrete structures are prone to cracking as a result of temperature and loading. Over time, the structure will accrue damage, resulting in resistance deterioration and, in severe situations, collapse [1,2]. As a result, it is critical to monitor the structure’s health.

At present, structural health monitoring is mostly accomplished by embedding conventional sensors in concrete structures such as resistance strain gages, optical fibers, and piezoelectric ceramics [3,4,5]. However, there is a major incompatibility issue between the sensors and the structure, such as incompatible acoustic impedance, uncooperative deformation, and poor interfacial adhesion [6].

In recent years, piezoelectric composites made of polymer and piezoelectric ceramic have received a significant amount of interest. Piezoelectric composites can sense varied stimuli in the surrounding environment and accomplish the objective of structural monitoring through the conversion of mechanical energy and electrical potential energy, gaining widespread use [7,8]. Li et al. employed cement as the matrix phase of a piezoelectric composite, which increased the composite’s compatibility with concrete [9]. However, the porosity of cement-based piezoelectric materials is high, the resistivity is easily affected by the environment, the sensitivity is low, combination with the piezoelectric phase is poor, and the piezoelectric performance is severely limited and cannot sufficiently meet the practical application [10,11,12,13]. In order to enhance the piezoelectric performance of composites, researchers have conducted extensive investigations. Two commonly utilized approaches for improving piezoelectric capabilities are: increasing the amount of the piezoelectric phase in the composite, and adding conductive elements to the base phase [14,15]. Fatemeh [16] and Qin [17] improved the piezoelectric properties of composites by adding inorganic piezoelectric fillers to the piezoelectric phase to increase the piezoelectric phase proportion. However, with the addition of inorganic fillers, the crystal structure of composites becomes unstable due to temperature effects. Gong [18] improved the piezoelectric and dielectric performance of a cement-based piezoelectric composite by incorporating conductive materials (such as carbon black and nanotubes). However, too much conductive material tends to be unevenly distributed, resulting in excessive local conductivity and breakdown of the piezoelectric composite during the polarization process. Kim [19] discovered that incorporating nanoparticles in piezoelectric composites can boost the power generation of flexible nanogenerators. However, because of the weak connection between ceramics and polymers and the high surface-to-volume ratio of nanoparticles, flexible nanogenerators (PCNPs) can easily form agglomerates in polymer nanofibers (NFs).

Furthermore, in order for the conductive phase to be properly compatible with the composite, the nanoparticles must go through a complicated surface functionalization procedure before they can be used. It is obvious from the literature above that existing strategies for improving piezoelectric properties have certain limitations. Increasing the amount of the piezoelectric phase in the composite can significantly boost the piezoelectric efficiency, but it will also reduce the composite’s compatibility with concrete. In addition, the incorporation of nanomaterials or conductive materials tends to agglomerate the piezoelectric composites or causes the piezoelectric composites to break down during polarization. The addition of additives to composites frequently necessitates particular processing for the additives to assure compatibility with the composite. This complicates and raises the expense of composite manufacture, which is not favorable to large-scale distribution. As a result, a low-cost method for properly balancing the piezoelectric characteristics and compatibility of piezoelectric composites is required.

When cement is used as the matrix material, the piezoelectric composite’s performance is limited by low early strength, high-temperature hydrolysis, high resistivity, and lack of density [20]. However, high early strength, adaptability, and low resistivity are all advantages of the alkali-activated fly ash/slag (AAFS) material, and AAFS also has a hydration product that is similar to cement. Instead of adding conductive material, replacing cement with materials with lower resistivity or minimizing the pores inside the composite could improve the piezoelectric and dielectric performance of the composite more effectively. Moreover, good compatibility could be achieved [21,22,23]. Therefore, a new solution is proposed in this paper. AAFS was used as a matrix material in the preparation of a 1-3-2 piezoelectric composite, and its properties were thoroughly investigated [24,25]. The sodium silicate modulus and SL/AF ratio are the most important factors that determine the properties of AAFS [26,27]. According to Lu Hui’s investigation of the conductivity of AAFS, the resistivity of slag and fly ash is lower than that of cement, and the resistivity changes with the SL/FA as well as the sodium silicate modulus [28]. Hence, in this paper, an experimental investigation was organized to find out the effect of the SL/FA ratio and sodium silicate modulus on the properties of a 1-3-2 alkali-based piezoelectric composite.

## 2. Experiment

### 2.1. Materials for Test Specimens

The matrix phase of the 1-3-2 piezoelectric composite in this paper was AAFS composed of slag and fly ash. The alkaline activator, which consisted of sodium hydroxide, distilled water, and sodium silicate, was used to activate the matrix material. The properties and chemical composition of the raw materials are listed in Table 1. Table 2 shows the specified content of the raw materials.

### 2.2. Specimen Preparation

#### 2.2.1. Calculation of Solution Modulus

In this experiment, the alkaline activator raw materials were mixed in a specific ratio to create an alkaline activator solution. The solution modulus, defined as the molar ratio of SiO_2_/Na_2_O, can have a significant impact on the hydration reaction [29]. The goal of this experiment was to adjust the modulus by changing the SS/SH molar ratio. The solution modulus was calculated according to GB/T 4209-2008 [30]. The following is the specific calculation formula:

(1) $$\left\{ \begin{array}{l} Q_{NO}=Q_{No}\times Q_{S}+\frac{2m_{NH}}{m_{NO}}Q_{NH}\\ M=\frac{Q_{No}}{w_{S}\times Q_{S}}\times \frac{m_{S}}{m_{NO}}\\ Q_{W}=w_{CW}\times Q_{P}-(1-w_{S}-w_{NO})\times Q_{S}-\frac{m_{W}}{2m_{NH}}Q_{NH} \end{array}\right.$$

where *Q_NO_*, *Q_S_*, *Q_NH_*, *Q_W_*, and *Q_P_* are the masses of Na_2_O, Na_2_SO_3_, NaOH, water, and powder (slag and fly ash), respectively; *m_NH_*, *m_NO_*, *m_S_*, and *m_W_* are the relative molecular masses of NaOH, Na_2_O, SO_2_, and water, respectively; *ω_s_* is the mass ratio of Na_2_SO_3_/SO_2_, where this experiment used 27.8%; *ω_NO_* is the mass ratio of Na_2_O/Na_2_SO_3_, where this experiment used 8.8%; *M* is the solution modulus; *ω_CW_* is the mass ratio of water/powder.

Assuming that the powder totals 100 g, the *ω_CW_* is 0.45, the M is 1.5, and the Na_2_O solid content in the Na_2_SO_3_ is 4%. According to formula (1), the mass of raw materials in the alkaline activator is shown in Table 3.

#### 2.2.2. The Ratio of Raw Materials

The experiments in this paper used an SL/FA ratio ranging from 0 to 1. The specimen designations are listed in Table 4. On the basis of best performance, the group with better test results was chosen for the subsequent study on the various modules. In general, as the amount of sodium hydroxide in the mixture increased, the hydroxide ion concentration increased, resulting in a faster dissolution of the raw materials [31]. This means that increasing the sodium hydroxide molarity affects the silica modulus of the alkaline liquid, which affects the alkaline activation process. The modulus of specimen group B is shown in Table 5.

#### 2.2.3. The Preparation Process

**Figure 1:**
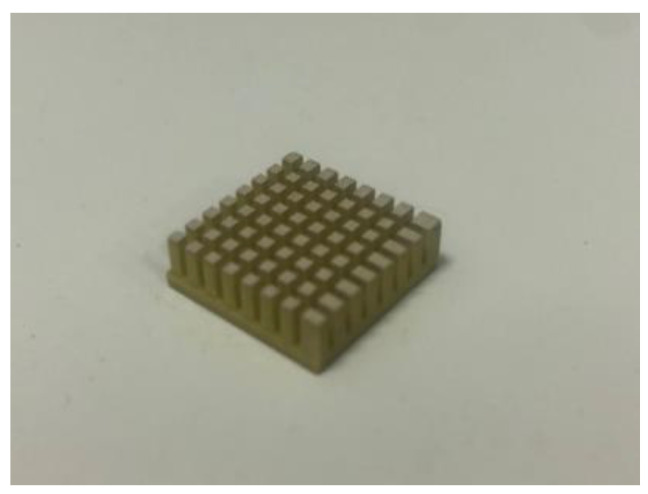
The skeleton of the specimen.

The cutting process: The cut-filling and arrange-filling techniques were used to create 1-3-2 AAFS-based piezoelectric composites in this paper [32]. The PZT5H was cut with a saw blade thickness of 0.5 mm using the SJY-400 cutter. The PZT5H ceramic was cut into a skeleton that included a base, grooves, and columns. The base was 1.0 mm thick, and the column and grooves were 4.0 mm tall. The column section was 1.0 × 1.0 mm^2^. The specimen was cleaned with an ultrasonic cleaning machine after it had been cut. The PZT5H was then installed in the mold for casting. Figure 1 illustrates the skeleton of the specimen.

The preparation of the activator: The NaOH and Na_2_·nSiO_2_ for the alkaline activator were prepared in a precise proportion. After combining the NaOH and Na_2_·nSiO_2_ with distilled water, the activator was placed in a plastic sealed bottle and cooled to room temperature for subsequent use.

**Figure 2:**
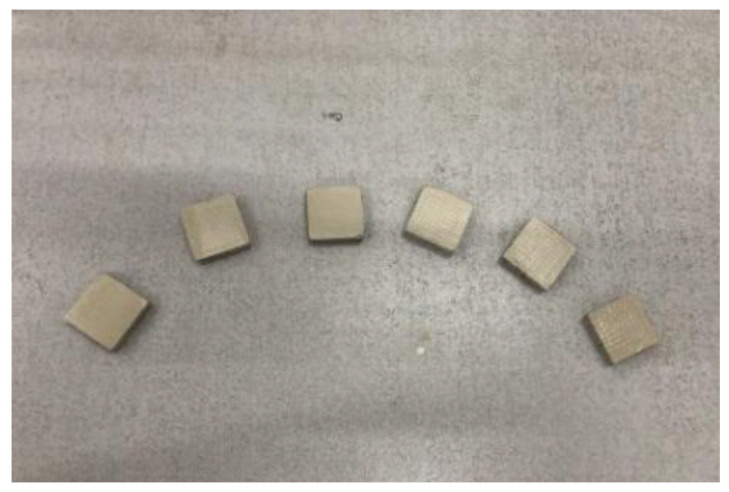
Photo of specimen.

**Figure 3:**
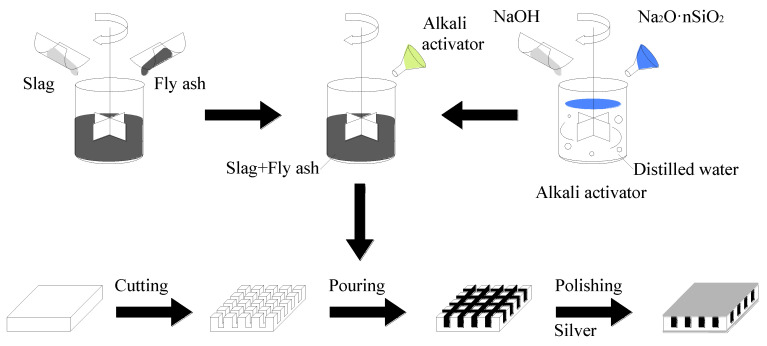
Dielectric properties.

The molding process: First, mix the fly ash, slag, and alkaline activator and stir them for 2 min. The mixture should then be poured into the ceramic grooves. Vibrate the specimen on the vibration table once the pouring is complete. Cure the specimen for 24 h at room temperature with cling film covering, and then de-mold and cure the specimen for 7 days in a standard maintenance box (temperature 28 °C, relative humidity 90%). After curing, grind and polish the top and bottom surfaces of the specimen before silver coating them. A 1-3-2 AAFS-based piezoelectric composite was fabricated using these processes. Figure 2 shows the finished specimens. The specimens’ compressive strength was measured using the INSTRON-5984 test machine. The compressive strength value exceeded 50 MPa, which is sufficient to meet the requirements of the sensor embedded in the structure. Figure 3 depicts the composite preparation flow chart.

### 2.3. Performance Test

The properties of the 1-3-2 AAFS-based piezoelectric composite were tested according to GB/T 3389-2008 [33]. The dielectric constant represents the dielectric’s performance for charge storage and loss, which is described by the relative dielectric constant *ε_r_* and dielectric loss *tanσ*, respectively. The Agilent 4294A Impedance Phase Analyzer was used to measure the specimen’s capacitance *C* and *tanσ* at a frequency of 1 kHz. Equation (2) can be used to calculate *ε_r_*, while Equation (3) can be used to calculate *tanσ*:

(2) $$\varepsilon_{r}=\frac{\varepsilon }{\varepsilon_{0}}=\frac{Ct}{A\varepsilon_{0}}$$

(3) $$\tan\;\sigma =\frac{1}{\omega CR}$$

where *t* and *A* are the thickness and electrode area of the specimen, respectively, *ε_o_* is the vacuum permittivity whose value is 8.85 × 10^−12^ F/m, and the *C* was measured by the capacitance tester at 1 kHz.

The piezoelectric property is a physical quantity that describes the coupling effect of elastic and electric polarization properties in piezoelectric materials. It is usually represented by the piezoelectric strain constant *d_33_* and piezoelectric voltage constant *g_33_*. In general, the higher the *d_33_*, the better the piezoelectric performance. The greater the *g_33_*, the greater the sensitivity of the composite material to receive the signal. The ZJ-3A *d_33_* Quasi-static Measuring Instrument was used to calculate the *d*_33_. Each specimen was measured four times at random locations on the top and bottom surfaces, and the average *d*_33_ value was calculated as the final value. The piezoelectric property is calculated as follows:

(4) $$d_{33}=(\frac{\partial S_{3}}{\partial E_{3}})T=(\frac{\partial D_{3}}{\partial T_{3}})E$$

(5) $$g_{33}=\frac{d_{33}}{\varepsilon_{r}\times \varepsilon_{0}}=\frac{d_{33}\times A}{Ct}$$

where S_3_ and E_3_ are the strain and electric field intensity, respectively, and D_3_ and T_3_ are the electric displacement and stress, respectively.

The electromechanical coupling performance of piezoelectric materials is a physical quantity that represents their ability to convert electric energy and mechanical energy. The planar electromechanical coupling coefficient *K_p_* and the thickness electromechanical coupling coefficient *K_t_* are commonly used to express the strength of the piezoelectric effect. The *K_p_*, resonance frequency *f*_s_, and anti-resonance frequency *f*_p_ were measured with an Agilent 4294A Impedance Phase Analyzer at 0–1 MHz. The following equation can be used to calculate *K_t_*:

(6) $$K_{t}^{2}=\frac{\pi f_{s}}{2f_{p}}tg(\frac{\pi }{2}\times \frac{f_{p}-f_{s}}{f_{s}})$$

The mechanical quality factor *Q_m_* represents the energy consumed by piezoelectric materials to overcome the internal friction. The Agilent 4294A Impedance Phase Analyzer was also used to measure its value. The following is the calculation formula:

(7) $$Q_{m}=\frac{2\pi W_{1}}{W_{2}}$$

where *W_1_* is the amount of mechanical energy stored at resonance within one cycle, and *W_2_* is the consumption of mechanical energy.

The acoustic impedance performance is a physical quantity that characterizes the acoustic characteristics of a medium. It can be calculated as follows:

(8) $$Z=2\rho_{c}f_{p}t_{\;}$$

where *ρ*_c_ is the density of the 1-3-2 AAFS-based piezoelectric composite.

Each designation group included three specimens, and the experiment results shown here are the averages of the three specimens’ test values.

## 3. Result and Discussion

### 3.1. Influence of SL/FA on the Properties of 1-3-2 AAFS-Based Piezoelectric Composite

#### 3.1.1. Influence of SL/FA on the Dielectric Properties

**Figure 4:**
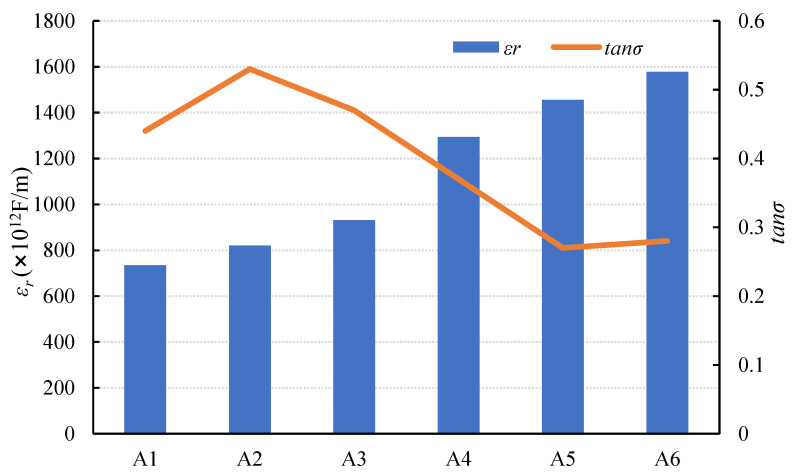
Changes in dielectric properties of specimens with SL/FA.

The values of *C* and *tanσ* were measured as described above, *ε_r_* was calculated using Equation (2), and the *ε_r_* and *tanσ* of each group (series A) are shown in Figure 4. Figure 4 shows that as the SL/FA ratio increased, *ε_r_* increased gradually, while *tanσ* decreased and then increased. As shown in Figure 4, composites with a slag content ranging from 40% to 60% exhibited a sharply increasing tendency for *ε_r_*.

The following are the reasons for this result. Because the *ε* of slag is greater than that of fly ash, the *ε_r_* of composites increases as the slag content increases. Because the ratio of fly ash is less than that of slag, the increase rate of *ε_r_* increases as the slag content increases from 40% to 60%. The *ε_r_* of alkali-activated piezoelectric composites is higher than that of several common 1-3-2 cement-based piezoelectric composites [34,35]. The feasibility of using AAFS as the matrix phase has been demonstrated, and the *ε_r_* can be adjusted by adjusting the SL/FA ratio.

**Figure 5:**
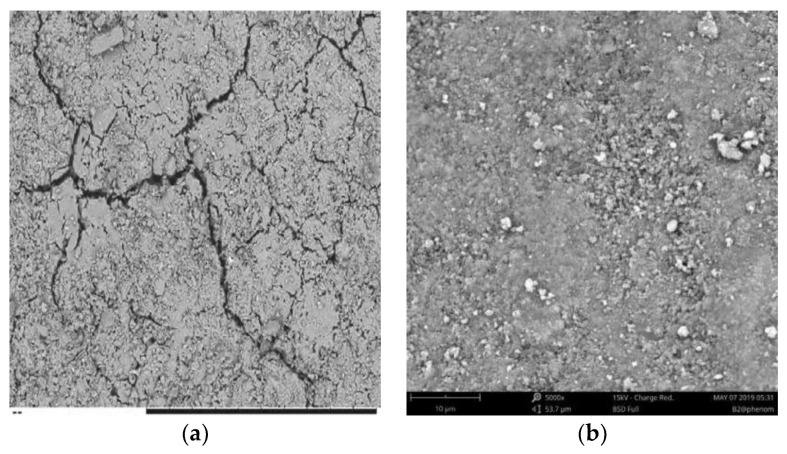
SEM photos of specimens: (**a**) slag content is 0%; (**b**) slag content is 10% [28].

According to previous research, the higher the SL/FA ratio, the higher the resistivity of AAFS and the lower the porosity, as shown in Figure 5 and Table 6 [28]. When only fly ash is used as the matrix phase’s raw material (i.e., the fly ash content is 100%), the slurry’s resistivity is lower due to the greater amount of free water. The value of tan*σ* increases when the slag content increases from 0% to 20% and decreases when the slag content increases from 20% to 100%. The reaction between the raw materials and activator was more thorough in the range of 0% to 20%, and more combined water was produced during the process, increasing the resistivity of the composite.

When the electrons migrated from the AAFS to the PZT5H, the higher resistivity led to lower conductivity, resulting in more energy dissipation. As a result, the value of *tanσ* increased. However, when the slag ratio grew, more gelling elements were formed, which filled the pores during the hydration process. As a result, the density of the composite was increased, while the porosity was reduced. Thus, when the slag ratio was in the range of 20% to 100%, *tanσ* showed a diminishing tendency.

#### 3.1.2. Influence of SL/FA on the Piezoelectric Properties

**Figure 6:**
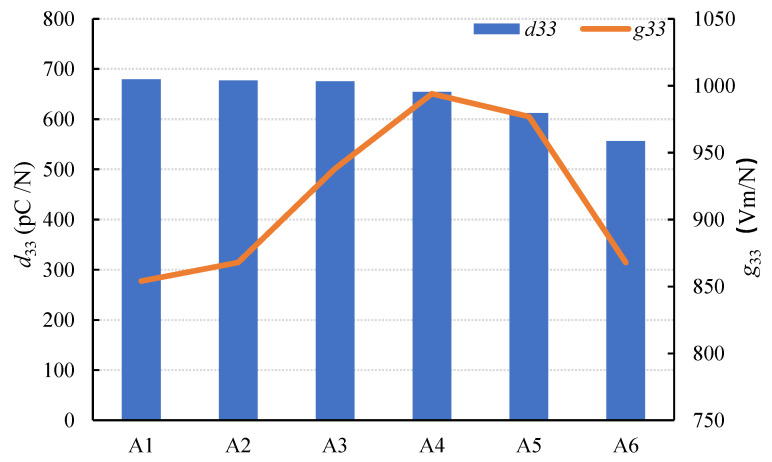
Changes in piezoelectric properties of specimens with SL/FA.

The piezoelectric properties of specimen series A are shown in Figure 6. The *d*_33_ was measured by the ZJ-3A *d*_33_ Quasi-static Measuring Instrument. The *g_33_* was calculated by Equation (5). Figure 6 shows that as the SL/FA ratio increased, *d_33_* gradually decreased, while *g_33_* increased and then decreased.

As shown in Table 6, the resistivity of the composite increased while the conductivity decreased as the SL/FA ratio increased. Because it is difficult for the electrons to migrate from the AAFS to the PZT5H, the piezoelectric effectiveness decreased. As a result, the value of *d_33_* decreased. The value of *g_33_*, on the other hand, increased first and then decreased. When the slag ratio was between 40% and 60%, the values of *d_33_* and *g_33_* increased. The coupling between the piezoelectric and dielectric performance, as well as the signal receiving sensitivity, was optimal in this case.

#### 3.1.3. Influence of SL/FA on the Electromechanical Coupling Performance and Mechanical Quality Factor

**Figure 7:**
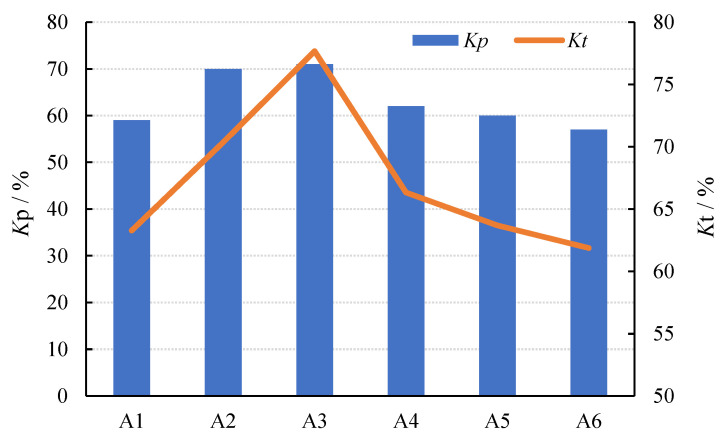
Changes in *K_t_* and *K_p_* of specimens with SL/FA.

**Figure 8:**
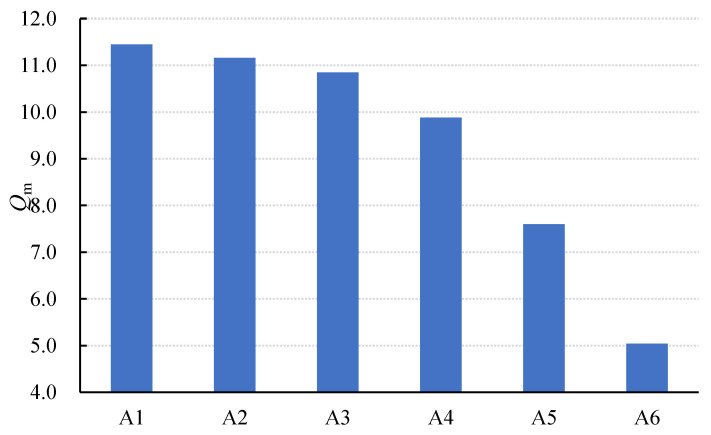
Changes in Qm of specimens with SL/FA.

As Figure 7 and Figure 8 show, the values of *K_t_* and *K_p_* increased first and then decreased with the increase in the SL/FA ratio, while the value of *Q_m_* showed a decreased trend. When the ratio of slag was 40%, *K_t_* and *K_p_* reached the highest values.

The following are the reasons for this result. The higher the SL/FA ratio, the lower the porosity, resulting in a closer connection between the PZT5H and AAFS. When the slag content increases from 0% to 40%, the coupling of the composites becomes more intense, resulting in an increase in *K_t_* and *K_p_*. When the slag content rises from 40% to 100%, it begins to exceed the content of fly ash. At this time, the decrease in *K_t_* and *K_p_* could be attributed to the slag’s stronger activity, which can quickly react with the activator and release a large amount of hydration heat, increasing the heat loss of the AAFS and lowering the electromechanical conversion efficiency of the composites [28].

The decrease in *Q_m_* is caused by an increase in resistivity. As shown in Table 6, as the slag content increases from 0% to 40%, the resistivity increases slightly but remains relatively low. As a result, the energy consumed by charge movement rises slightly, while *Q_m_* falls with a relatively low trend. On the contrary, when the slag content exceeds 40%, the resistivity of the AAFS rapidly increases, resulting in a rapid increase in the energy consumed by the charge movement in the composites, causing greater energy consumption of the charge movement and a rapid decrease in *Q_m_*.

#### 3.1.4. Influence of SL/FA on the Acoustic Impedance Performance

The *ρ_c_* and *f_p_* of the composites were measured with the electronic balance, and the *Z* was calculated by Equation (8). The results are shown in Table 7.

As shown in Table 7, the value of Z increased from 8.44 Mrayl to 11.01 Mrayl as the SL/FA ratio increased. When the slag ratio ranged from 0% to 40%, the composite’s Z value was most similar to that of the cement (9.0 Mrayl), allowing for non-destructive testing of the concrete. The value of Z is proportional to *ρ_c_*, *f_p_*, and *t*, according to Equation (8). Under the assumption of a constant thickness, Z is primarily determined by the composite’s *ρ_c_* and *f_p_*. As shown in Table 7, the higher the SL/FA ratio, the lower the porosity, and the higher the c and *f_p_*. The Z of the composites showed an increasing trend as *f*_p_ and *ρ*_c_ increased.

### 3.2. Influence of the Sodium Silicate Modulus

Considering the overall piezoelectric and dielectric performance, the composite had an optimal performance when the content of slag was 40%. Therefore, based on the slag content of 40%, the composites were prepared with a sodium silicate modulus from 1.0 to 1.5 and then numbered as B1–B6, respectively, as shown in Table 5.

#### 3.2.1. Influence of the Sodium Silicate Modulus on Dielectric Properties

**Figure 9:**
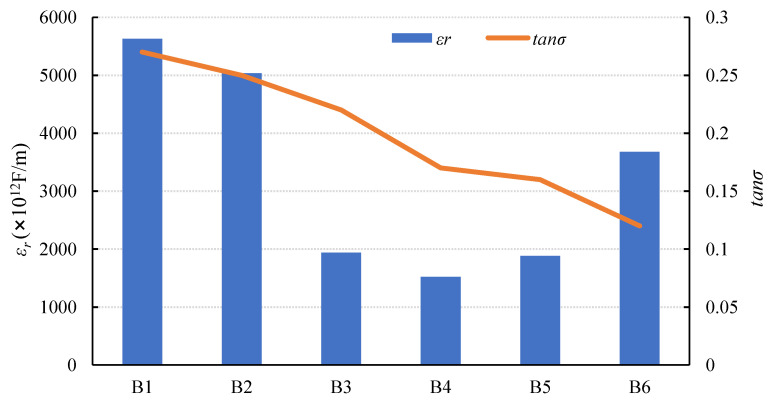
Changes in dielectric properties with modulus.

Figure 9, Table 8, and Equation (2) show that as the sodium silicate modulus increases, *ε_r_* increases first and then decreases due to *C* variation, whereas *tanσ* shows a decreasing trend. The following are the reasons for this. In general, the lower the sodium silicate modulus, the higher the OH^-^ concentration, the faster the hydration reaction rate, and the more hydration heat there is [36,37], resulting in a higher *tanσ*. The value of *tanσ* continues to decrease when the modulus is in the range of 1.3–1.5, which could be due to the lower amount of more distilled water in the activator. Distilled water primarily participates in cation transport and further hydration in the later curing process during the reaction process. Therefore, more distilled water results in the lower resistivity of the AAFS [28] and *tanσ*.

The addition of sodium silicate solution increased the amount of (OH)_2_SiO_2_^2−^ and (OH)SiO_3_^3−^ in the composite, causing the concentration of ${\mathrm{Al}(\mathrm{OH})}_{4}^{-}$ to decrease. As a result, particle dissolution was accelerated, and the overall polymerization reaction was boosted. According to previous research, as the sodium silicate modulus increases, the accumulated heat release of AAFS decreases [38,39,40]. This could be because the lower modulus provides more OH-, which is destructive to the slag/fly ash vitreous structure. Therefore, in this paper, AAFS had the greatest accumulated heat release when the sodium silicate modulus was 1.0. Additionally, the composite exerted less heat as the sodium silicate modulus increases, and thus the value of tan decreased continuously.

#### 3.2.2. Influence of the Sodium Silicate Modulus on Piezoelectric Properties

**Figure 10:**
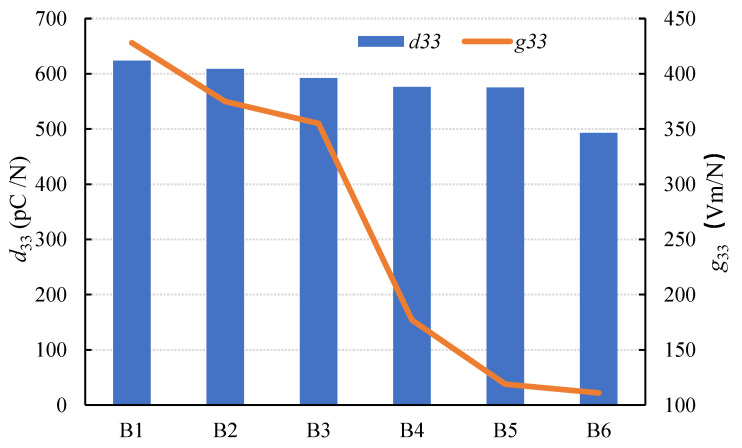
Changes in piezoelectric properties with modulus.

The results of the piezoelectric properties are shown in Figure 10. It can be seen that with the increase in the modulus, *d_33_* and *g_33_* show a decreasing trend. When the modulus was 1.0–1.3, the value of *d_33_*, *g_33_* reached a higher value. In this case, the coupling between piezoelectric performance and dielectric performance, as well as the signal receiving sensitivity, was optimal.

**Figure 11:**
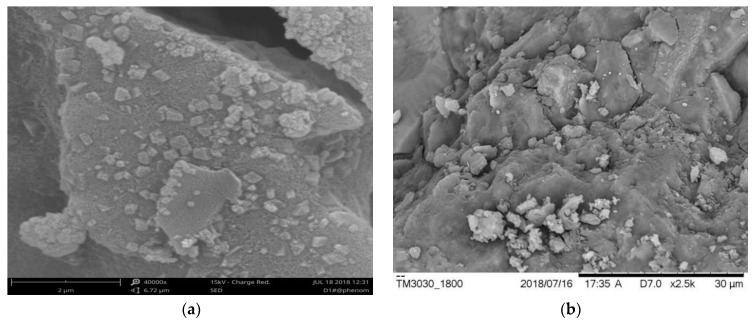
SEM photos of specimens: (**a**) modulus is 1.1; (**b**) modulus is 1.3 [28].

When the sodium silicate solution modulus is low, crystallization in the composite occurs more easily. When the modulus is too high, the alkali concentration decreases, which is detrimental to aluminosilicate dissolution and results in an insufficient composite strength [41]. As shown in Figure 11, the sodium silicate modulus ranged from 1.0 to 1.3, indicating a higher content of NaOH and a faster rate of hydration, which is advantageous for forming C-S-H gelling. The formed C-S-H gelling would fill the pores between the PZT5H and AAFS, increasing the density of the composite. As a result, the buffer effect of the pores was reduced under pressure, and the strain of the composite was reduced. According to Equation (4), as the conductive ion decreases, the piezoelectric effect of the composite decreases, and thus the value of *d*_33_ decreases.

When the modulus varied between 1.3 and 1.5, the increase in AAFS resistivity resulted in a greater overall resistivity of the composite. The electronic migration from the AAFS to the PZT5H was more difficult, which resulted in a *d_33_* decrease. This could be because the activator contained less distilled water when the sodium silicate modulus was high. Throughout the process, water was involved in the transportation of positive ions as well as lateral hydration during the maintenance process. On the other hand, the value of *g*_33_ showed a general downward trend. This could be because the rate of hydration was faster when the sodium silicate modulus was low. As the sodium silicate modulus increased, the rate of hydration slowed, resulting in a continuous decrease in the *g*_33_ value.

#### 3.2.3. Influence of the Sodium Silicate Modulus on Electromechanical Coupling Performance and Mechanical Quality Factor

**Figure 12:**
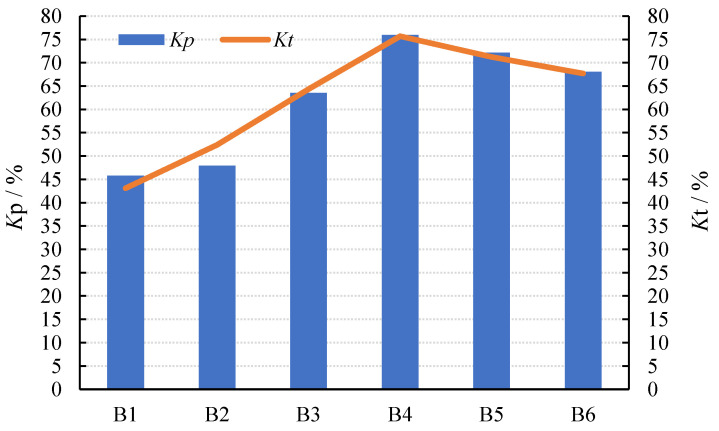
Electromechanical coupling performance.

**Figure 13:**
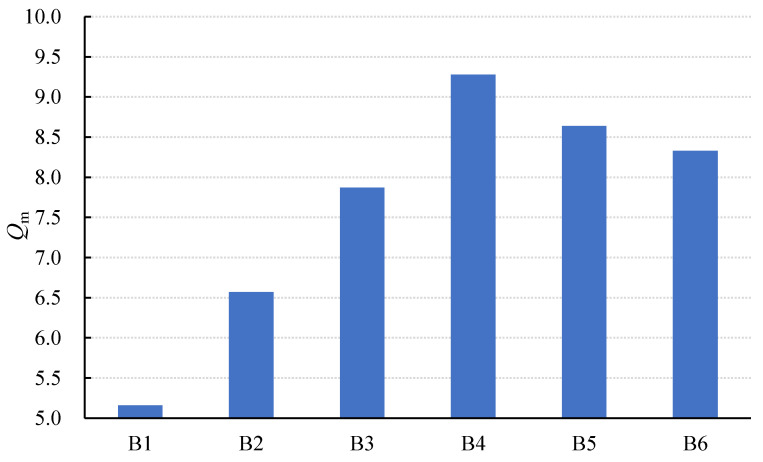
Mechanical quality factor.

As Figure 12 and Figure 13 show, the value of *K_t_* and *K_p_* increased first and then decreased with the increase in the modulus, while *Q_m_* decreased first and then increased. When the modulus was 1.3, *K_t_* and *K_p_* reached the highest values, and *Q_m_* reached the lowest value at the same time.

The variation in *K*_t_ and *K*_p_ was caused by porosity. When the modulus increased between 1.0 and 1.3, the alkalinity decreased [36,37]. At the same time, the main product of the reaction was C-S-H gelling, which allowed the Ca^2+^ in the slag to efficiently enhance the bonding of the AAFS, resulting in the lower porosity of the composites, as shown in Figure 12. Additionally, the composites’ coupling became more intense, resulting in an increase in *K_t_* and *K_p_*. When the modulus reached 1.3–1.5, the particle’s inorganic polymerization could not fully react. This may result in the rapid formation of a protective film on the surface of the alkali-activated material, obstructing the subsequent hydration reaction, increasing the porosity, and weakening the coupling effect. As a result, the value of *K_t_* and *K_p_* decreased.

The porosity of the composite was related to the value of *Q_m_*. The porosity between AAFS and PZT5H decreased as the modulus was increased from 1.0 to 1.3. The lower the porosity, the lower the attenuation of sound energy. As a result, the *Q_m_* value increased. When the sodium silicate modulus ranged from 1.3 to 1.5, particle decomposition and hydration occurred quickly. A protective film would quickly form on the surface of the hydration product, impeding the lateral hydration process. As a result, the porosity increased, while the *Q_m_* value decreased.

#### 3.2.4. Influence of the Sodium Silicate Modulus on Electromechanical Coupling Performance and Mechanical Quality Factor

The test results of specimens are shown in Table 9.

**Figure 14:**
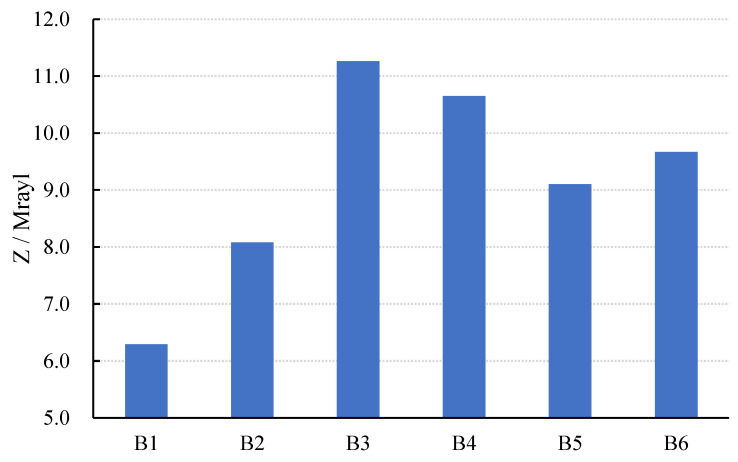
Changes in *Z* of specimens with modulus.

As shown in Figure 14, the *Z* value increased continuously as the sodium silicate modulus increased from 1.0 to 1.3, while it decreased as the sodium silicate modulus increased from 1.3 to 1.5. This could be due to the composite having a higher *f_p_* when the sodium silicate modulus fluctuates between 1.0 and 1.3, and a lower *f_p_* when the sodium silicate modulus fluctuates between 1.3 and 1.5. The Z value of specimens B5 and B6 was closest to that of the concrete for a sodium silicate modulus in the range of 1.0 to 1.3, which is advantageous for fabricating the sensor for NDT used in the concrete structure.

It can be seen from Figure 14 that the *Z* value continuously rose when the sodium silicate modulus increase from 1.0 to 1.3, while the Z value decreased as the sodium silicate modulus increased from 1.3 to 1.5. This may be due to the higher *f_p_* when the sodium silicate modulus fluctuates in the range of 1.0 to 1.3, the lower fluctuation range of *ρ_c_*, and the lower when the sodium silicate modulus increases from 1.3 to 1.5. For the sodium silicate modulus in the range of 1.0 to 1.3, the *Z* value of specimens B5 and B6 was closest to that of the concrete, which is beneficial for fabricating the sensor for NDT used in the concrete structure.

## 4. Conclusions

The fabrication and properties of a 1-3-2 AAFS-based piezoelectric composite with different SL/FA ratios and sodium silicate moduli were investigated in this study. Following this, the best mixtures were proposed. The following are the main conclusions that can be drawn from the experimental results:

(1) As the SL/FA ratio increased, the relative dielectric constant *ε_r_* showed an overall increasing trend, whereas dielectric loss tanσ increased first and then decreased. When the slag content was greater than 40%, the dielectric performance of the composites improved. When the slag content was between 40% and 60%, the composite had a higher piezoelectric strain constant *d*_33_ and piezoelectric voltage constant *g*_33_. The composite in this case had an optimal piezoelectric performance and a higher electromechanical coupling effect.
(2) As the SL/FA ratio increased, the mechanical quality factor *Q_m_* decreased due to the increasing resistivity. The coupling of the composite intensified as the SL/FA ratio grew, and the planar electromechanical coupling coefficient *K_p_* and the thickness electromechanical coupling coefficient *K_t_* both increased. However, when the content of slag exceeded 40%, the reaction between the slag and activator was rapid, increasing the heat loss of the AAFS. *K_t_* and *K_p_* decreased as a result of this. When the slag content was 40%, *K_t_* and *K_p_* reached their maximum, and the composite demonstrated a good electromechanical coupling effect. The acoustic impedance Z of the composite was close to that of the concrete, which was advantageous for NDT on the concrete structure. In terms of overall performance, the composite performed best when the slag content was 40%. As a result, the effect of the sodium silicate modulus was investigated further under the assumption of a 40% slag content.
(3) As the sodium silicate modulus increased, *ε_r_* increased first and then decreased due to the variation in capacitance *C*. In general, a higher sodium silicate modulus would slow the hydration reaction and reduce the hydration heat. Thus, tan*σ* exhibited an overall decreasing trend. The composites had a higher *d_33_* value when the sodium silicate modulus fluctuated between 1.0 and 1.3. Furthermore, the decline in *g_33_* in this range was very small. As a result, the composite performed best in terms of piezoelectricity when the sodium silicate modulus was between 1.0 and 1.3.
(4) When the sodium silicate modulus was increased from 1.0 to 1.3, the bonding of AAFS improved, while the porosity of the composite decreased. However, when the sodium silicate modulus exceeded 1.3, a protective film rapidly formed on the surface of the alkali-activated material, impeding the lateral hydration process. Therefore, *K_t_* and *K_p_* increased and subsequently decreased, as did *Q*_m_. When the sodium silicate modulus was 1.3, *Q_m_*, *K_t_*, and *K_p_* reached their maximum values, and the composite demonstrated excellent electromechanical coupling.
(5) In particular, the specimen with a 40% slag content and a sodium silicate modulus of 1.3 had the highest *Q_m_*, electromechanical coupling factor, and piezoelectric performance.
(6) Compared with cement-based piezoelectric composites, 1-3-2 alkali-based piezoelectric composites have better piezoelectric and mechanical properties, a higher degree of tunability, and better compatibility with concrete. The sensors made from them have an excellent performance and low cost and can be widely used for concrete structure health monitoring.

## Figures and Tables

**Table 1:** Material properties.

Materials	Properties of Materials
Slag	S95 level waste residue, ignition loss −1.5%, density 2.84 g/cm^3^, dielectric constant ε 7.80, chemical composites are shown in Table 2.
Fly Ash	Class I power plant waste (Class I waste residue), ignition loss 1.5%, density 2.81 g/cm^3^, dielectric constant ε 5.58, chemical composites are shown in Table 2.
Sodium Hydroxide	Analytical purity, NaOH ≥ 96%, Na_2_CO_3_ ≤ 1.2%, NaCl ≤ 2.5, Fe_2_O_3_ ≤ 0.008.
Sodium Silicate	Industrial purity, density 1.38 g/cm^3^, SiO_2_ and Na_2_O contents are 27.8% and 8.8%, respectively.
Water	Deionized water.
PZT5H	Niobium magnesium lead zirconate titanate, density 7.6 g/cm^3^, processed into a size of 22 × 22 × 5 as the specimen.

Pozzolans: SL: slag; FA: fly ash; FA: fly ash; SH: sodium hydroxide; SS: sodium silicate.

**Table 2:** Chemical composition of the base phase material.

Oxide Content (%)	SiO_2_ (%)	Al_2_O_3_ (%)	CaO (%)	Fe_2_O_3_ (%)	MgO (%)	SO_3_ (%)	Na_2_O (%)	K_2_O (%)	Others (%)
Fly Ash	55.00	15.10	13.36	5.96	2.83	0.68	1.6	3.76	1.71
Slag	34.36	16. 89	38.13	0.36	6.23	2.30	0.24	0.41	1.08

**Table 3:** The mass of raw materials in the alkaline activator.

*Q_S_* (g)	*Q_NH_* (g)	*Q_W_* (g)	Alkaline Activator (g)
21.10	2.82	31.44	55.35

**Table 4:** Designations of SL/FA.

Number	A1	A2	A3	A4	A5	A6
Fly Ash (%)	100	80	60	40	20	0
Slag (%)	0	20	40	60	80	100

**Table 5:** Modulus of specimen group B.

Number	B1	B2	B3	B4	B5	B6
Modulus	1.0	1.1	1.2	1.3	1.4	1.5

**Table 6:** Changes in resistivity of specimens with SL/FA [28].

SL/FA	0:100	20:80	40:60	60:40	80:20	100:0
Resistivity	0.488	2.812	4.093	15.228	17.014	25.800

**Table 7:** Changes in *Z* of specimens with SL/FA.

Number	*ρ_c_* (×10^6^ kg/m^3^)	*f_p_* (kHz)	*t* (×10^−3^ m)	*Z* (Mrayl)
A1	3.57	236.50	5.00	8.44
A2	3.60	258.25	5.00	9.29
A3	3.64	265.07	5.00	9.67
A4	3.69	271.23	5.00	10.02
A5	3.86	275.11	5.00	10.62
A6	3.90	282.50	5.00	11.01

**Table 8:** Changes in *C* with modulus.

Number	B1	B2	B3	B4	B5	B6
*C* (pF)	4822	4311	1661	1300	1610	3151

**Table 9:** Changes in *Z* of specimens with modulus.

Number	*ρ_c_* (×10^6^ kg/m^3^)	*f_p_* (kHz)	*t* (×10^−3^ m)	*Z* (Mrayl)
B1	3.87	162.50	5.0	6.29
B2	3.77	214.05	5.0	8.08
B3	3.77	298.75	5.0	11.26
B4	3.67	290.00	5.0	10.65
B5	3.74	243.25	5.0	9.10
B6	3.67	263.50	5.0	9.67
